# Cerebral Metabolic Differences Associated with Cognitive Impairment in Parkinson’s Disease

**DOI:** 10.1371/journal.pone.0152716

**Published:** 2016-04-11

**Authors:** Yilin Tang, Jingjie Ge, Fengtao Liu, Ping Wu, Sisi Guo, Zhenyang Liu, Yixuan Wang, Ying Wang, Zhengtong Ding, Jianjun Wu, Chuantao Zuo, Jian Wang

**Affiliations:** 1 Department of Neurology, Huashan Hospital, Fudan Universtiy, 12 Wulumuqi Zhong Road, Shanghai, 200040, China; 2 PET center, Huashan Hospital, Fudan University, 518 East Wuzhong Road, Shanghai, 200235, China; Biomedical Research Foundation, UNITED STATES

## Abstract

**Purpose:**

To characterize cerebral glucose metabolism associated with different cognitive states in Parkinson’s disease (PD) using ^18^F-fluorodeoxyglucose (FDG) and Positron Emission Tomography (PET).

**Methods:**

Three groups of patients were recruited in this study including PD patients with dementia (PDD; n = 10), with mild cognitive impairment (PD-MCI; n = 20), and with no cognitive impairment (PD-NC; n = 30). The groups were matched for age, sex, education, disease duration, motor disability, levodopa equivalent dose and Geriatric Depression Rating Scale (GDS) score. All subjects underwent a FDG-PET study. Maps of regional metabolism in the three groups were compared using statistical parametric mapping (SPM5).

**Results:**

PD-MCI patients exhibited limited areas of hypometabolism in the frontal, temporal and parahippocampal gyrus compared with the PD-NC patients (p < 0.01). PDD patients had bilateral areas of hypometabolism in the frontal and posterior parietal-occipital lobes compared with PD-MCI patients (p < 0.01), and exhibited greater metabolic reductions in comparison with PD-NC patients (p < 0.01).

**Conclusions:**

Compared with PD-NC patients, hypometabolism was much higher in the PDD patients than in PD-MCI patients, mainly in the posterior cortical areas. The result might suggest an association between posterior cortical hypometabolism and more severe cognitive impairment. PD-MCI might be important for early targeted therapeutic intervention and disease modification.

## Introduction

Cognitive impairment is known to be a common non-motor symptom in individuals with Parkinson’s disease (PD), and ultimately 80% of PD patients will progress to Parkinson’s disease with dementia (PDD) [1]. Cognitive impairment of a lesser severity is designated as mild cognitive impairment (PD-MCI) [2], which is conceptualized as a transitional stage between normal cognition and dementia. Increasing evidence suggests that PD-MCI might be a powerful predictor for the development of dementia [3].

Radionuclide brain imaging, by means of SPECT and PET, can help in understanding the pathophysiological basis of cognitive deficits. Previous FDG-PET studies have revealed a prevalence of posterior parietal and occipital hypometabolism in PDD subjects [4–5]. Recent studies have also demonstrated that specific patterns of cortical and subcortical metabolic abnormalities are associated with PD-MCI patients, characterized by reduced metabolism in the frontal and temporo-parieto-occipital regions [6]. PET imaging could also be a useful tool for evaluating brain metabolic changes over time. A longitudinal study evaluating glucose metabolism in non-demented PD patients found that those who converted to dementia several years later had a reduction in FDG uptake in visual association and posterior cingulate cortices at baseline compared with controls, concluding that these metabolic reductions could represent an early predictor of dementia [7]. Other research has also demonstrated that significant hypometabolism in the precunei and temporal areas may be associated with the onset of significant cognitive decline [8].

However, most previous studies have focused mainly on comparing the cerebral metabolism of PD patients with controls, the regional cerebral glucose metabolism features among the different cognitive states in PD, particularly PDD and PD-MCI, were poorly elucidated. Additionally, regional cerebral glucose metabolism in PD-MCI has not yet been investigated in a Chinese cohort. Therefore, in this study we used FDG-PET to characterize the metabolic differences between different cognitive states in PD.

## Materials and Methods

### Subjects

Sixty patients with PD were recruited from the Department of Neurology, Huashan Hospital, Fudan University, between March, 2011 and November, 2014. Before entering the study, all subjects were screened and clinically examined by two senior investigators of movement disorders. Based on the UK Brain Bank criteria [9], a diagnosis of PD was made in all subjects if the patients had ‘pure’ parkinsonism without a history of known causative factors such as encephalitis or neuroleptic treatment, and did not have supranuclear gaze abnormalities or ataxia. Cases with any history of cerebrovascular disease, metabolic disease, head injury, severe psychiatric illness, or with abnormal findings on MRI or CT were excluded from the study.

All participants provided written informed consent in accordance with the Declaration of Helsinki. All aspects of the study were approved by the Human Studies Institutional Review Board, Huashan Hospital, Fudan University.

### Clinical and neuropsychological evaluation

The patients were off anti-parkinsonian medications for at least 12h before clinical assessment. The severity and stage of the patient’s parkinsonism was evaluated using the Unified Parkinson’s Disease Rating Scale motor (UPDRS-III) subscore and the modified Hoehn and Yahr stage. To standardize data on medication use, we converted dosages of PD medications to total daily levodopa equivalent doses. None of the patients were treated with benzodiazepines, neuroleptics or antidepressants.

The patients underwent neuropsychological examination while on their routine medications. Global cognition was evaluated using the Mini Mental State Examination (MMSE) [10]. Depression was rated using the Geriatric Depression Rating Scale (GDS) [11]. Five specific cognitive domains were assessed by a complete neuropsychological battery. Attention and working memory were assessed utilizing the Symbol Digit Modality Test (SDMT) [12] and Trail Making Test A (TMT-A) [13]. Executive function was assessed utilizing Stroop Color-Word Test (CWT) [14] and Trail Making Test B (TMT-B) [13]. Language was assessed utilizing Boston Naming Test (BNT) and Animal Fluency Test (AFT) [15]. Memory was assessed utilizing Auditory Verbal Learning Test (AVLT) [16] and delayed recall of the Rey-Osterrieth Complex Figure Test [17]. Visuospatial function was assessed utilizing Clock Drawing Test [18] and copy task of Rey-Osterrieth Complex Figure test [17] (S1 Table).

The clinical diagnostic criteria for dementia in PD were applied to diagnose dementia in the present study [19–20]. MCI was diagnosed according to the recommendations of the Movement Disorder Society (MDS) Task Force 2012 by Level 2 [21]. The performance on a cognitive test was considered abnormal if the score was 1.5 SDs below the norm. Impairment on at least 2 neuropsychological tests, represented by either 2 tests showing impairment in 1 cognitive domain or 1 test showing impairment in 2 different cognitive domains, was required. PD patients who did not fulfill the criteria for PD-MCI or PDD were classified as PD-NC.

### PET imaging

Patients underwent a FDG-PET study and neuropsychological evaluation within 3 months. Before FDG PET imaging, the patients were asked to fast for at least 6 h, but had free access to water. Before injection of the radiopharmaceutical agent, blood glucose was checked and confirmed to be <120 mg/dl in all cases. PET scans were performed with a Siemens Biograph 64 PET/CT (Siemens, Germany) in three-dimensional (3D) mode. A CT transmission scan was first performed for attenuation correction. The scan was started 45 min after an intravenous bolus injection of 185 MBq of FDG and lasted for 10 min. Hanning filters were used during image reconstruction with filtered-backprojection, giving a transaxial and axial cut-off frequency of 0.5. As no arterial blood sampling was taken in this clinical imaging protocol, we could not measure absolute glucose metabolism in our subjects. Instead, we used radioactivity count images to measure changes in relative regional glucose metabolism. All studies in patients and normal individuals were performed in a resting state in a quiet and dimly lit room.

### Imaging Processing

Preprocessing of imaging data was performed by SPM5 software implemented in Matlab7.4.0 (Mathworks Inc, Sherborn, MA). Scans from each subject were spatially normalized into Montreal Neurological Institute (MNI) brain space with linear and nonlinear three-dimensional transformations. The normalized PET images were then smoothened by a Gaussian filter of 10 mm FWHM (reduced Full Width at Half Maximum) over a 3D space to increase signal to noise ratio for statistical analysis.

### Data Analysis

PET imaging data were analyzed by using the SPM5 software as described previously [22]. To characterize metabolic activity in PD groups and PD patients compared with controls, we performed a group comparison by using a two-sample t-test according to the general linear model at each voxel. Mean signal differences over the whole brain were removed by analysis of covariance in each individual subject.

To evaluate the results, we set the peak threshold at P<0.01 (uncorrected) over whole brain regions with an extent threshold of 80 voxels (corresponding to a tissue volume of 640mm^3^). Significant regions were localized by Talairach–Daemon software (Research Imaging Center, University of Texas Health Science Center, San Antonio, TX, USA). The SPM maps for altered glucose metabolism were overlaid on a standard T1-weighted magnetic resonance imaging (MRI) brain template in stereotaxic space.

One-way analysis of variance was applied to test for differences in clinical characteristics and neuropsychological scores among PD groups (PD-NC, PD-MCI, PDD) and post hoc Scheffe was used for multiple comparison. Differences among groups for sex were evaluated with χ^2^. All analyses were performed using the SPSS software (SPSS for Windows, version 19.0; SPSS Inc., Chicago, IL, USA) and considered significant for P<0.05.

## Results

### Clinical data

The subjects included 60 PD patients, 30 were classified as PD-NC, 20 as PD-MCI and 10 as PDD. No significant differences were observed between groups in terms of age, sex, years of education, duration of disease, Hoehn and Yahr stage, UPDRS-III score, levodopa equivalent dose or GDS score. The detailed demographic and clinical profiles are shown in Table 1 and S2 Table.

**Table 1:** Demographic and clinical profiles of subjects.

	PD-NC	PD-MCI	PDD	p Value^a^
**No. of subjects**	30	20	10	—
**Age, y**	61.9±6.3	61.9±6.7	61.4±10.5	0.982
**F/M**	14/16	10/10	3/7	0.564
**Education, y**	12.9±3.0	11.2±3.6	11.5±4.6	0.203
**Disease duration, y**	3.6±3.2	5.7±4.5	5.2±3.9	0.154
**Hoehn and Yahr stage**	1.8±0.8	2.1±1.1	2.5±0.8	0.097
**UPDRS-III score**^**b**^	23.0±8.1	30.0±17.4	30.7±11.9	0.089
**Levodopa equivalent dose (mg/day)**	190.0±227.1	307.0±336.6	255.0±117.3	0.302
**GDS score**	11.6±7.3	11.2±7.0	13.0±8.3	0.829
**Blood glucose (mg/dl)**^**c**^	94.4±9.4	94.3±10.7	91.8±9.4	0.75

PD-NC, Parkinson’s disease with no cognitive impairment; PD-MCI, Parkinson’s disease with mild cognitive impairment; PDD, Parkinson’s disease with dementia; UPDRS, Unified Parkinson’s Disease Rating Scale; GDS, Geriatric Depression Rating Scale.

The data are presented as mean ± SD.

^a^ Analysis of variance with the exception of chi-square for gender.

^b^ Off-state motor ratings according to the UPDRS (motor section).

^c^ Blood glucose was checked before injection of the radiopharmaceutical agent.

### Cognitive and behavioral profiles

Compared with the PD-NC and PD-MCI patients, PDD patients had poorer scores in all neuropsychological tests. PD-MCI patients in turn had lower scores than PD-NC patients for all tests (Table 2 and S3 Table). The cognitive domains affected in PD-MCI patients were as follows: 1 patient (5%) had only the attention domain affected; 7 patients (35%) had two domains affected; 7 patients (35%) had three domains affected; 4 patients (20%) had four domains affected; and 1 patient (5%) had five domains affected.

**Table 2:** Neuropsychological testing in Parkinson’s disease patients.

Cognitive test	PD-NC	PD-MCI	PDD	p Value	Post hoc significance
**MMSE**	28.5±1.7	28.4±1.3	23.2±2.3	<0.001	[D<N^c^] [D<M^c^]
**Attenion and working memory**					
SDMT	37.4±7.1	24.3±9.1	24.3±16.8	<0.001	[M<N^c^] [D<N^b^]
TMT-A (s)	57.8±14.3	71.2±19.9	96.7±53.8	0.001	[D>N^b^]
**Executive function**					
CWT-C time (s)	69.7±13.3	84.7±24.3	122.4±37.9	<0.001	[M>N^a^] [D>N^c^] [D>M^c^]
CWT-C right	48.5±2.2	44.6±6.9	40.3±7.0	<0.001	[M<N^a^] [D<N^c^]
TMT-B (s)	152.0±34.6	200.8±61.3	245.8±91.7	<0.001	[M>N^a^] [D>N^c^]
**Language**					
BNT	24.6±3.2	22.3±2.7	21.1±3.9	0.005	[M<N^a^] [D<N^a^]
AFT	17.1±3.5	16.2±3.3	13.3±3.1	0.012	[D<N^a^]
**Memory**					
AVLT-delay recall	5.9±2.7	3.7±2.2	2.4±2.0	<0.001	[M<N^a^] [D<N^b^]
AVLT-T	29.7±10.1	22.7±7.4	13.7±6.8	<0.001	[M<N^a^] [D<N^c^] [D<M^a^]
CFT-delay recall	17.0±6.2	12.3±5.9	8.3±7.2	0.001	[M<N^a^] [D<N^b^]
**Visuospatial function**					
CFT	34.3±2.1	30.6±5.6	21.7±13.4	<0.001	[D<N^c^] [D<M^b^]
CDT	23.0±5.1	18.4±6.7	11.4±8.6	<0.001	[M<N^a^] [D<N^c^] [D<M^a^]

PD-NC, Parkinson’s disease with no cognitive impairment; PD-MCI, Parkinson’s disease with mild cognitive impairment; PDD, Parkinson’s disease with dementia; MMSE, Mini Mental State Examination; SDMT, Symbol Digit Modality Test; TMT, Trail Making Test; CWT, Stroop Color-Word Test; BNT, Boston Naming Test; AFT, Animal Fluency Test; AVLT, Auditory Verbal Learning Test; CFT, the Rey-Osterrieth Complex Figure Test; CFT, Clock Drawing Test; N, PD-NC; M, PD-MCI; D, PDD. p Value represents the significance level of the analysis of variance performed for each test across the three groups. The data are presented as mean ± SD.

^a^ p < 0.05

^b^ p < 0.01

^c^ p < 0.001

### Regional differences of cerebral metabolism

**Fig 1:**
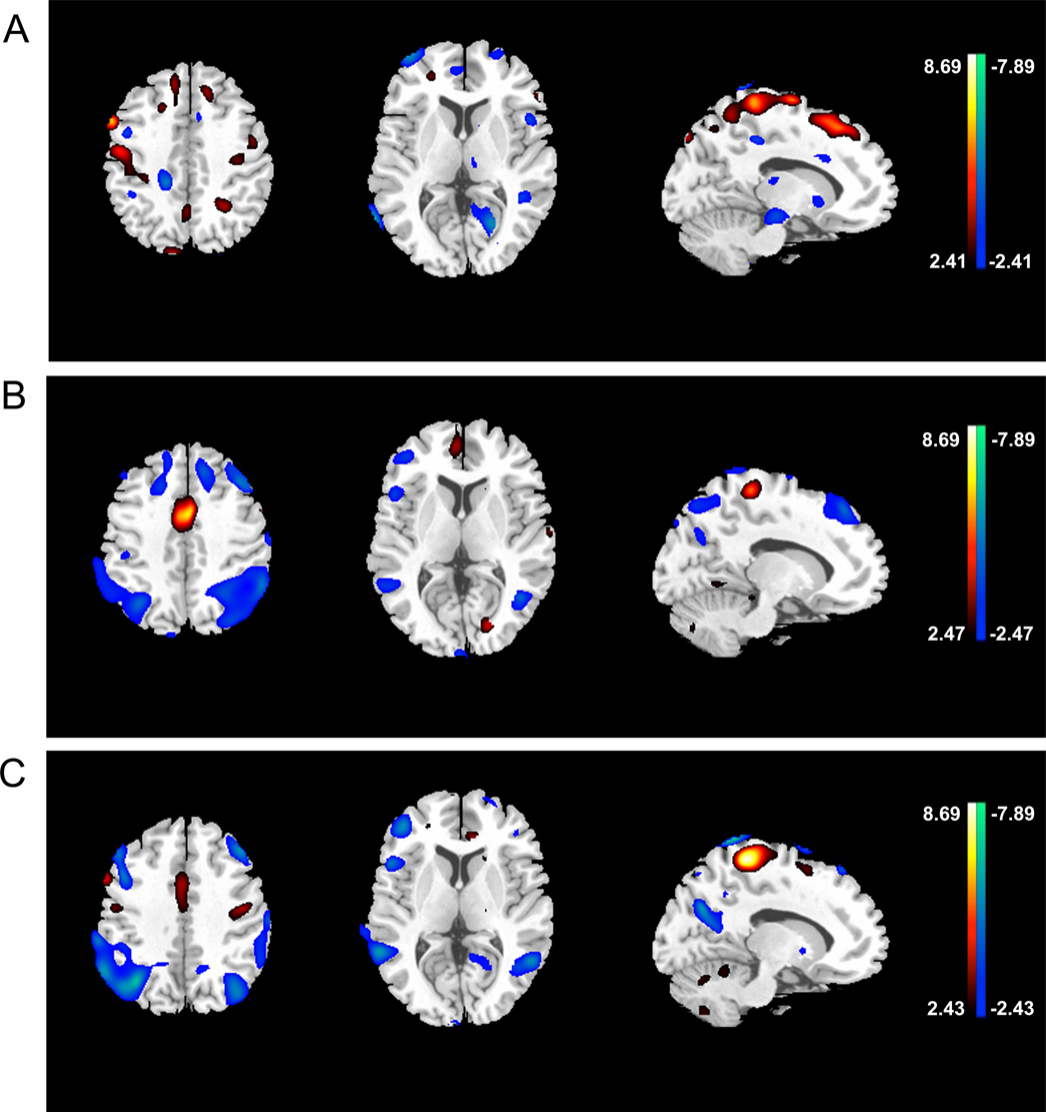
Group comparison of regional metabolic changes between PD groups utilizing voxel-based statistical parametric mapping analysis. Metabolic increases are displayed using a red–yellow scale and declines are displayed using a blue–purple scale. Both displays were superimposed on a single-subject MRI brain template and thresholded at p = 0.01 (uncorrected). (A) PD-MCI VS. PD-NC, (B) PDD VS. PD-MCI, (C) PDD VS. PD-NC.

Regions with significant differences across the PDD, PD-MCI, and PD-NC groups are presented in Fig 1 and Table 3. Compared with controls, PD-MCI group revealed limited areas of hypometabolism in the right superior frontal gyrus, right precentral gyrus, left superior temporal gyrus, left posterior cingulate and left parahippocampal gyrus, and limited hypermetabolism in the left postcentral gyrus, left paracentral lobule and right precentral gyrus (p < 0.01). PDD group had FDG uptake reduction in the right superior frontal gyrus, left precentral gyrus, left parietal lobule, right angular gyrus, left supramarginal gyrus, left precuneus and cuneus, associated with increased metabolism in the left cingulate gyrus compared with PD-MCI group (p < 0.01). PDD group showed relative hypometabolism in the right frontal lobe, right inferior parietal lobule, right supramarginal gyrus, bilateral middle temporal gyrus, left posterior cingulate, bilateral precuneus and left cuneus, associated with hypermetabolism in right paracentral lobule compared with PD-NC patients (p < 0.01).

**Table 3:** Brain regions with significant metabolic differences between PD groups (p<0.01 uncorrected).

	Regions	BA	MNI coordinate^a^			Z_max_	Cluster size (mm^3^)
			x	y	z		
PD-MCI VS.PD-NC^b^							
Increased metabolism	Lt postcentral gyrus	5	-18	-46	70	4.07	32600
	Lt paracentral lobule	4	-10	-44	74	3.91	
	Rt precentral gyrus	4	18	-38	72	3.63	
Decreased metabolism	Lt superior temporal gyrus	22	-62	-4	-2	3.81	1024
	Lt posterior cingulate	30	-24	-70	10	3.36	976
	Rt precentral gyrus	6	44	22	38	3.15	1376
	Rt superior frontal gyrus	10	42	62	0	3.14	1064
	Lt parahippocampal gyrus	19	-20	-46	-10	3.07	1304
PDD VS. PD-MCI							
Increased metabolism	Lt cingulate gyrus	24	-2	2	42	3.09	2184
Decreased metabolism	Lt precentral gyrus	6	-66	-16	34	4.19	1400
	Rt superior frontal gyrus	8	20	26	50	3.35	2184
	Lt supramarginal gyrus	40	-60	-46	28	3.32	3568
	Lt inferior parietal lobule	40	-58	-50	40	2.93	
	Lt superior parietal lobule	7	-28	-56	40	2.62	
	Rt angular gyrus	39	34	-64	38	3.24	1376
	Lt precuneus	31	-12	-68	-20	2.93	1400
	Lt cuneus	17	-20	-82	4	2.42	648
PDD VS. PD-NC							
Increased metabolism	Rt paracentral lobule	6	12	-36	66	4.12	30928
Decreased metabolism	Lt middle temporal gyrus		-60	-48	-4	3.61	2736
	Rt inferior frontal gyrus	9	44	10	32	3.39	1440
	Rt superior frontal gyrus	8	44	16	56	3.24	1592
	Rt middle frontal gyrus	9	48	30	42	2.82	
	Rt inferior parietal lobule	39	36	-66	40	3.23	5432
	Rt supramarginal gyrus	40	62	-56	32	3.16	
	Lt cuneus	18	-24	-72	14	3.02	1624
	Lt posterior cingulate	30	-10	-62	14	2.69	
	Rt precuneus	7	14	-70	30	2.97	1176
	Rt precuneus	31	18	-64	20	2.91	
	Lt precuneus	19	-40	-78	34	2.96	2120
	Rt middle temporal gyrus	21	60	-50	4	2.96	1272

BA, Brodmann area; MNI, Montreal Neurological Institute; PD-NC, Parkinson’s disease with no cognitive impairment; PD-MCI, Parkinson’s disease with mild cognitive impairment; PDD, Parkinson’s disease with dementia; Lt, Left; Rt, Right.

^a^ MNI standard space.

^b^ Survived at uncorrected P < 0.01, extent threshold = 80 voxels (640 mm^3^).

## Discussion

The current study found that early cognitive decline in Parkinson’s disease, defined as PD-MCI, was already associated with limited areas of hypometabolism predominantly in the frontal and temporal cortices compared with PD-NC patients. PDD patients exhibited more widespread hypometabolism, mainly located in the posterior parietal-occipital regions, compared with PD-MCI patients, and exhibited greater metabolic reductions in comparison with cognitively unimpaired PD patients.

In our study, the cortical hypometabolism observed in the PD-MCI group was limited, mainly located in the frontal and temporal lobes relative to PD-NC patients. Previous studies in PD-MCI patients showed hypometabolism predominantly in the frontal [23–26], parietal [23, 25–27], and occipital [27] cortices or more extensive involvement of temporo-parieto-occipital regions [6]. The discrepancy was probably due to the lack of a cohesive definition of PD-MCI in the literature, as changes observed could be associated with varying severity of impairment depending on the study criteria used. Here we diagnosed patients with PD-MCI using MDS level II category guidelines [21].This is thought to be a more stringent diagnostic criterion that allows PD-MCI patients with lower levels of cognitive impairment to be included. Moreover, hypermetabolism in limited areas of frontal lobes was also observed in this study, presumably related to frontal compensation [28]. Our research in Chinese patients has revealed PD is associated with significant metabolic reduction since the early cognitive decline across populations of different ethnicity.

The exploration of cerebral glucose metabolism in PDD started in 1985 [29]. In subsequent studies of severely affected PDD subjects, hypometabolism has been detected in the posterior cingulate, parietal, and temporal association regions, with a lesser involvement of the frontal cortex, when compared with nondemented PD patients and healthy controls [4–5]. However, less information is available on comparison between PDD and PD-MCI patients. Here we found that PDD patients had extensive bilateral areas of hypometabolism in the frontal and posterior parietal-occipital lobes compared with PDMCI patients, and exhibited greater metabolic reductions in comparison with PDNC patients. The results of our study were consistent with a cross-sectional study in which PDD was found to be characterized by a more expansive cerebral hypometabolism than PD-MCI, predominantly in the posterior cortical areas [25]. The posterior cortical activity changes may reflect cholinergic denervation secondary to loss of nucleus basalis of Meynert afferents [30–31]. Supporting this hypothesis, cholinergic dysfunction has been reported to be much greater in PDD than in nondemented PD subjects [32–33]. The PDD subjects in this study also had significantly hypometabolism involving the frontal lobes compared with PD-MCI patients. The involvement of the frontal lobes may reflect that PDD subjects have more impaired prefrontal dopamine signaling. It has been proposed that mesocortical dopaminergic projections can influence cognitive function [34]. The decrease in dopamine concentrations was greater in demented than in non-demented patients with PD, which suggests a role for the degeneration of mesocortical dopaminergic system in the development of dementia [35–36]. Overall, the anterior and posterior cortical hypometabolism observed in PDD patients may in part be due to mixed effects of dopaminergic and cholinergic denervation [31].

The PD-MCI patients in our study showed limited areas of hypometabolism compared with PD-NC patients, whereas PDD patients exhibited widespread hypometabolism mainly in posterior areas compared with PD-MCI patients. This result might suggest an association between more severe cognitive impairment and posterior cortical hypometabolism. Furthermore, a 5-year follow-up study of cognitive from a cohort of incidental PD patients showed that early cognitive deficits related to posterior but not frontal cortex predicted more rapid cognitive decline and early dementia [37]. This hypothesis was supported by a prospective cohort study concluding that PDD is heralded by hypometabolism in posterior cortices [7]. Besides, the PD-MCI group, in which the hypometabolism of the posterior areas was limited in our study, might be an important stage for future studies concerning the delay or prevention of PDD. A recent study found that hypometabolism exceeds atrophy in some brain regions in PD patients with cognitive impairment [38]. The authors speculated that the non-atrophic hypometabolism areas might be considered ‘metabolic penumbra’ where cell loss is putatively reversible. Moreover, some investigations have reported that PD-MCI might be an unstable state with reversion to normal cognitive status at follow-up, even when the diagnosis of PD-MCI was based on comprehensive cognitive test batteries. The Norwegian ParkWest study reported a 25% reversal of PD-MCI to normal cognition over a 3-year period [39]. Broeders et al. reported less than 10% of PD-MCI cases reverting to normal cognition at 5 years [40]. Thus, given the relatively slight brain metabolic changes and probable fluctuating status, PD-MCI should be given more focus for early targeted therapeutic intervention and disease modification.

As previously mentioned, a limitation of this study was the cross-sectional study with a relatively small patient sample size, not allowing the comparison between baseline PET findings and the clinical outcome. Future longitudinal studies in larger group of patients with longer clinical follow-up are required to confirm these findings. A previous study [25] was limited by the lack of matched factors between the patient groups such as age, depression and motor severity. There were no such significant group differences in age, sex, years of education, duration of disease, levodopa equivalent dose, GDS scores and motor severity in our study. Therefore we believe our results would not have been significantly affected by these factors. Another strength of the study was the comprehensive battery of neuropsychological tests, with two or more tests for each cognitive domain, meeting a more stringent diagnostic criterion set by MDS.

## Conclusions

Our results might be useful in identifying metabolic differences associated with different cognitive status in PD. For the first time we detected the hypometabolism predominantly in the frontal and temporal cortices in Chinese PD-MCI patients compared with PD-NC patients. Hypometabolism was much higher in the PDD patients than in PD-MCI patients, mainly in the posterior cortical areas. Ongoing follow-up will enable us to better evaluate such brain metabolic changes as ideal biomarkers for assessing the severity of cognitive impairment in PD or predicting the risk of developing PDD.

## Supporting Information

S1 Table Detailed descriptions of the neuropsychological test. (DOCX)

S2 Table P-values of Scheffe’s test for differences in clinical characteristics between the PD groups. (DOCX)

S3 Table P-values of Scheffe’s test for differences in neuropsychological scores between the PD groups. (DOCX)

## References

[1] Hely MA, Reid WG, Adena MA, Halliday GM, Morris JG. The Sydney multicenter study of Parkinson's disease: the inevitability of dementia at 20 years. Mov Disord 2008;23(6):837–44. 10.1002/mds.21956 18307261

[2] Caviness JN, Driver-Dunckley E, Connor DJ, Sabbagh MN, Hentz JG, Noble B, et al Defining mild cognitive impairment in Parkinson's disease. Mov Disord 2007;22(9):1272–7. 17415797 10.1002/mds.21453

[3] Kehagia AA, Barker RA, Robbins TW. Neuropsychological and clinical heterogeneity of cognitive impairment and dementia in patients with Parkinson's disease. Lancet Neurol 2010;9(12):1200–13. 10.1016/S1474-4422(10)70212-X 20880750

[4] Vander BT, Minoshima S, Giordani B, Foster NL, Frey KA, Berent S, et al Cerebral metabolic differences in Parkinson's and Alzheimer's diseases matched for dementia severity. J Nucl Med 1997;38(5):797–802. 9170449

[5] Peppard RF, Martin WR, Carr GD, Grochowski E, Schulzer M, Guttman M, et al Cerebral glucose metabolism in Parkinson's disease with and without dementia. Arch Neurol 1992;49(12):1262–8. 1449406 10.1001/archneur.1992.00530360060019

[6] Hosokai Y, Nishio Y, Hirayama K, Takeda A, Ishioka T, Sawada Y, et al Distinct patterns of regional cerebral glucose metabolism in Parkinson's disease with and without mild cognitive impairment. Movement Disorders 2009;24(6):854–862. 10.1002/mds.22444 19199357

[7] Bohnen NI, Koeppe RA, Minoshima S, Giordani B, Albin RL, Frey KA, et al Cerebral Glucose Metabolic Features of Parkinson Disease and Incident Dementia: Longitudinal Study. Journal of Nuclear Medicine 2011;52(6):848–855. 10.2967/jnumed.111.089946 21571793 PMC7486964

[8] Tard C, Demailly F, Delval A, Semah F, Defebvre L, Dujardin K, et al Hypometabolism in Posterior and Temporal Areas of the Brain is Associated with Cognitive Decline in Parkinson's Disease. J Parkinsons Dis 2015;5(3):569–74. 10.3233/JPD-150583 26406137

[9] Hughes AJ, Daniel SE, Kilford L, Lees AJ. Accuracy of clinical diagnosis of idiopathic Parkinson's disease: a clinico-pathological study of 100 cases. J Neurol Neurosurg Psychiatry 1992;55(3):181–4. 1564476 10.1136/jnnp.55.3.181 PMC1014720

[10] Katzman R, Zhang MY, Ouang-Ya-Qu, Wang ZY, Liu WT, Yu E, et al A Chinese version of the Mini-Mental State Examination; impact of illiteracy in a Shanghai dementia survey. J Clin Epidemiol 1988;41(10):971–8. 3193141 10.1016/0895-4356(88)90034-0

[11] Yesavage JA, Brink TL, Rose TL, Lum O, Huang V, Adey M, et al Development and validation of a geriatric depression screening scale: a preliminary report. J Psychiatr Res 1982;17(1):37–49. 7183759 10.1016/0022-3956(82)90033-4

[12] Sheridan LK, Fitzgerald HE, Adams KM, Nigg JT, Martel MM, Puttler LI, et al Normative Symbol Digit Modalities Test performance in a community-based sample. Arch Clin Neuropsychol 2006;21(1):23–8. 16139470 10.1016/j.acn.2005.07.003

[13] Zhao Q, Guo Q, Li F, Zhou Y, Wang B, Hong Z. The Shape Trail Test: application of a new variant of the Trail making test. PLoS One 2013;8(2):e57333 10.1371/journal.pone.0057333 23437370 PMC3577727

[14] Steinberg BA, Bieliauskas LA, Smith GE, Ivnik RJ. Mayo's Older Americans Normative Studies: Age- and IQ-Adjusted Norms for the Trail-Making Test, the Stroop Test, and MAE Controlled Oral Word Association Test. Clin Neuropsychol 2005;19(3–4):329–77. 16120535 10.1080/13854040590945210

[15] Lucas JA, Ivnik RJ, Smith GE, Ferman TJ, Willis FB, Petersen RC, et al Mayo's Older African Americans Normative Studies: norms for Boston Naming Test, Controlled Oral Word Association, Category Fluency, Animal Naming, Token Test, WRAT-3 Reading, Trail Making Test, Stroop Test, and Judgment of Line Orientation. Clin Neuropsychol 2005;19(2):243–69. 16019707 10.1080/13854040590945337

[16] Guo Q, Zhao Q, Chen M, Ding D, Hong Z. A comparison study of mild cognitive impairment with 3 memory tests among Chinese individuals. Alzheimer Dis Assoc Disord 2009;23(3):253–9. 10.1097/WAD.0b013e3181999e92 19812468

[17] Caffarra P, Vezzadini G, Dieci F, Zonato F, Venneri A. Rey-Osterrieth complex figure: normative values in an Italian population sample. Neurol Sci 2002;22(6):443–7. 11976975 10.1007/s100720200003

[18] Guo Q, Fu JH, Yuan J. A study of validity of a new scoring system of clock drawing test. Chin J Neurol 2008;41(4):234–237.

[19] Emre M, Aarsland D, Brown R, Burn DJ, Duyckaerts C, Mizuno Y, et al Clinical diagnostic criteria for dementia associated with Parkinson's disease. Movement Disorders 2007;22(12):1689–1707. 17542011 10.1002/mds.21507

[20] Dubois B, Burn D, Goetz C, Aarsland D, Brown RG, Broe GA, et al. Diagnostic procedures for Parkinson's disease dementia: Recommendations from the movement disorder society task force. Movement Disorders 2007;22(16):2314–2324. 18098298 10.1002/mds.21844

[21] Litvan I, Goldman JG, Tröster AI, Schmand BA, Weintraub D, Petersen RC, et al Diagnostic criteria for mild cognitive impairment in Parkinson's disease: Movement Disorder Society Task Force guidelines. Movement Disorders 2012;27(3):349–356. 10.1002/mds.24893 22275317 PMC3641655

[22] Zuo C, Ma Y, Sun B, Peng S, Zhang H, Eidelberg D, et al Metabolic imaging of bilateral anterior capsulotomy in refractory obsessive compulsive disorder: an FDG PET study. J Cereb Blood Flow Metab 2013;33(6):880–7. 10.1038/jcbfm.2013.23 23443174 PMC3677106

[23] Huang C, Mattis P, Perrine K, Brown N, Dhawan V, Eidelberg D. Metabolic abnormalities associated with mild cognitive impairment in Parkinson disease. Neurology 2008;70(16 Pt 2):1470–7. 10.1212/01.wnl.0000304050.05332.9c 18367705 PMC4454398

[24] Pappata S, Santangelo G, Aarsland D, Vicidomini C, Longo K, Bronnick K, et al Mild cognitive impairment in drug-naive patients with PD is associated with cerebral hypometabolism. Neurology 2011;77(14):1357–62. 10.1212/WNL.0b013e3182315259 21940621

[25] Garcia-Garcia D, Clavero P, Gasca Salas C, Lamet I, Arbizu J, Gonzalez-Redondo R, et al Posterior parietooccipital hypometabolism may differentiate mild cognitive impairment from dementia in Parkinson’s disease. European Journal of Nuclear Medicine and Molecular Imaging 2012;39(11):1767–1777. 10.1007/s00259-012-2198-5 22872307

[26] Lyoo CH, Jeong Y, Ryu YH, Rinne JO, Lee MS. Cerebral Glucose Metabolism of Parkinson’s Disease Patients with Mild Cognitive Impairment. European Neurology 2010;64(2):65–73. 10.1159/000315036 20606450

[27] Nobili F, Abbruzzese G, Morbelli S, Marchese R, Girtler N, Dessi B, et al. Amnestic mild cognitive impairment in Parkinson's disease: A brain perfusion SPECT study. Movement Disorders 2009;24(3):414–421. 10.1002/mds.22381 19235928

[28] Narayanan NS, Rodnitzky RL, Uc EY. Prefrontal dopamine signaling and cognitive symptoms of Parkinson’s disease. Reviews in the Neurosciences 2013;24(3). 10.1515/revneuro-2013-0004 PMC3836593 23729617

[29] Kuhl DE, Metter EJ, Benson DF, Ashford JW, Riege WH, Fujikawa DG, et al. Similarities of Cerebral Glucose-Metabolism in Alzheimers and Parkinsonian Dementia. Journal of Nuclear Medicine 1985;26(5):P69–P69.

[30] Perry RH, Tomlinson BE, Candy JM, Blessed G, Foster JF, Bloxham CA, et al Cortical cholinergic deficit in mentally impaired Parkinsonian patients. Lancet 1983;2(8353):789–90. 10.1016/s0140-6736(83)92317-6 6137620

[31] Hilker R, Thomas AV, Klein JC, Weisenbach S, Kalbe E, Burghaus L, et al Dementia in Parkinson disease: functional imaging of cholinergic and dopaminergic pathways. Neurology 2005;65(11):1716–22. 16344512 10.1212/01.wnl.0000191154.78131.f6

[32] Bohnen NI, Kaufer DI, Ivanco LS, Lopresti B, Koeppe RA, Davis JG, et al Cortical cholinergic function is more severely affected in parkinsonian dementia than in Alzheimer disease: an in vivo positron emission tomographic study. Arch Neurol 2003;60(12):1745–8. 14676050 10.1001/archneur.60.12.1745

[33] Shinotoh H, Namba H, Yamaguchi M, Fukushi K, Nagatsuka S, Iyo M, et al In vivo mapping of brain cholinergic function in Parkinson's disease and progressive supranuclear palsy. Adv Neurol 2001;86:249–55. 11553984

[34] Narayanan NS, Land BB, Solder JE, Deisseroth K, DiLeone RJ. Prefrontal D1 dopamine signaling is required for temporal control. Proceedings of the National Academy of Sciences 2012;109(50):20726–20731. 10.1073/pnas.1211258109 PMC3528521 23185016

[35] Scatton B, Javoy-Agid F, Rouquier L, Dubois B, Agid Y. Reduction of cortical dopamine, noradrenaline, serotonin and their metabolites in Parkinson's disease. Brain Res 1983;275(2):321–8. 6626985 10.1016/0006-8993(83)90993-9

[36] Emre M. Dementia associated with Parkinson's disease. Lancet Neurol 2003;2(4):229–37. 12849211 10.1016/s1474-4422(03)00351-x

[37] Williams-Gray CH, Evans JR, Goris A, Foltynie T, Ban M, Robbins TW, et al The distinct cognitive syndromes of Parkinson's disease: 5 year follow-up of the CamPaIGN cohort. Brain 2009;132(Pt 11):2958–69. 10.1093/brain/awp245 19812213

[38] Gonzalez-Redondo R, Garcia-Garcia D, Clavero P, Gasca-Salas C, Garcia-Eulate R, Zubieta JL, et al Grey matter hypometabolism and atrophy in Parkinson's disease with cognitive impairment: a two-step process. Brain 2014;137(8):2356–2367. 24951642 10.1093/brain/awu159 PMC4610189

[39] Pedersen KF, Larsen JP, Tysnes OB, Alves G. Prognosis of mild cognitive impairment in early Parkinson disease: the Norwegian ParkWest study. JAMA Neurol 2013;70(5):580–6. 10.1001/jamaneurol.2013.2110 23529397

[40] Broeders M, de Bie RM, Velseboer DC, Speelman JD, Muslimovic D, Schmand B. Evolution of mild cognitive impairment in Parkinson disease. Neurology 2013;81(4):346–52. 10.1212/WNL.0b013e31829c5c86 23794682

