# Supplementary material for: Cerebral Metabolic Differences Associated with Cognitive Impairment in Parkinson’s Disease
Source: PLoS One. 2016 Apr 11;11(4):e0152716. doi: 10.1371/journal.pone.0152716 (PMC4827825; doi:10.1371/journal.pone.0152716)
Supplement: S1 Table — (DOCX) [file pone.0152716.s001.docx]

**S1 Table. Detailed descriptions of the neuropsychological test.**

| **Cognitive domains** | **Tests** | **Descriptions** |
| --- | --- | --- |
| **Attenion and working memory** | Symbol Digit Modality Test (SDMT) [1] | Numbers ranging from 1 to 9, with each digit matched to a different geometrical symbol. Write down the digit according to the symbol as quickly as possible. |
|  | Trail Making Test-A (TMT-A) [2] | Scan and connect all numbers distributed in a spatial array. |
| **Executive function** | Stroop Color-Word Test (CWT) [3] | Identify the color of print in which a color name is written rather than the reading of the name itself. (Max score=110) |
|  | Trail Making Test-B (TMT-B) [2] | Scan and connect alternating numbers and letters distributed in a spatial array. |
| **Language** | Boston Naming Test (BNT) [4] | Name 30 line drawings of common objects shown sequentially, each within 20sec. (Max score=30) |
|  | Animal Fluency Test (AFT) [4] | Name as many animals as possible within one minute |
| **Memory** | Auditory Verbal Learning Test (AVLT) [5] | A list of 12 items is presented three times, each followed by free recall testing. After an interference test lasting five minutes, free recall of the list for the fourth time (short delayed free recall). After another twenty minutes, free and cued recall for the fifth time (long delayed free and cued recall), and choose the right items from a total of 24 (recognition). (Max score of recognition=24, the rest=12) |
|  | delayed recall of the Rey-Osterrieth Complex Figure Test (CFT-delay recall) [6] | 20-25 minutes after copying, recall the complex line-drawing figure. |
| **Visuospatial function** | Clock Drawing Test (CDT) [7] | Draw a clock and mark the time 1:50. (Max score=30) |
|  | copy task of Rey-Osterrieth Complex Figure test (CFT) [6] | Copy one complex line-drawing figur without reminding later recall. (Max score=36) |

[1] Sheridan LK, Fitzgerald HE, Adams KM, Nigg JT, Martel MM, Puttler LI*, et al.*. Normative Symbol Digit Modalities Test performance in a community-based sample. Arch Clin Neuropsychol 2006;21(1):23-8.

[2] Zhao Q, Guo Q, Li F, Zhou Y, Wang B, Hong Z. The Shape Trail Test: application of a new variant of the Trail making test. PLoS One 2013;8(2):e57333.

[3] Steinberg BA, Bieliauskas LA, Smith GE, Ivnik RJ. Mayo's Older Americans Normative Studies: Age- and IQ-Adjusted Norms for the Trail-Making Test, the Stroop Test, and MAE Controlled Oral Word Association Test. Clin Neuropsychol 2005;19(3-4):329-77.

[4] Lucas JA, Ivnik RJ, Smith GE, Ferman TJ, Willis FB, Petersen RC*, et al.*. Mayo's Older African Americans Normative Studies: norms for Boston Naming Test, Controlled Oral Word Association, Category Fluency, Animal Naming, Token Test, WRAT-3 Reading, Trail Making Test, Stroop Test, and Judgment of Line Orientation. Clin Neuropsychol 2005;19(2):243-69.

[5] Guo Q, Zhao Q, Chen M, Ding D, Hong Z. A comparison study of mild cognitive impairment with 3 memory tests among Chinese individuals. Alzheimer Dis Assoc Disord 2009;23(3):253-9.

[6] Caffarra P, Vezzadini G, Dieci F, Zonato F, Venneri A. Rey-Osterrieth complex figure: normative values in an Italian population sample. Neurol Sci 2002;22(6):443-7.

[7] Guo Q, Fu JH, Yuan J. A study of validity of a new scoring system of clock drawing test. Chin J Neurol 2008;41(4):234-237.
