# Supplementary material for: Cerebral Metabolic Differences Associated with Cognitive Impairment in Parkinson’s Disease
Source: PLoS One. 2016 Apr 11;11(4):e0152716. doi: 10.1371/journal.pone.0152716 (PMC4827825; doi:10.1371/journal.pone.0152716)
Supplement: S2 Table — (DOCX) [file pone.0152716.s002.docx]

**S2 TABLE. P values of Scheffe’s test for differences in clinical characteristics between the PD groups.**

|  | **PD-NC** | **PD-MCI** | **PDD** | **p Value^a^** | **p Value^b^** | **p Value^c^** |
| --- | --- | --- | --- | --- | --- | --- |
| **No. of subjects** | 30 | 20 | 10 | — | — | — |
| **Age, y** | 61.9±6.3 | 61.9±6.7 | 61.4±10.5 | 1.000 | 0.987 | 0.982 |
| **F/M** | 14/16 | 10/10 | 3/7 | 0.817 | 0.297 | 0.356 |
| **Education, y** | 12.9±3.0 | 11.2±3.6 | 11.5±4.6 | 0.237 | 0.967 | 0.558 |
| **Disease duration, y** | 3.6±3.2 | 5.7±4.5 | 5.2±3.9 | 0.183 | 0.957 | 0.510 |
| **Hoehn and Yahr stage** | 1.8±0.8 | 2.1±1.1 | 2.5±0.8 | 0.513 | 0.517 | 0.110 |
| **UPDRS-III score^d^** | 23.0±8.1 | 30.0±17.4 | 30.7±11.9 | 0.161 | 0.990 | 0.249 |
| **Levodopa equivalent dose (mg/day)** | 190.0±227.1 | 307.0±336.6 | 255.0±117.3 | 0.307 | 0.893 | 0.823 |
| **GDS score** | 11.6±7.3 | 11.2±7.0 | 13.0±8.3 | 0.984 | 0.832 | 0.887 |
| **Blood glucose (mg/dl)^e^** | 94.4±9.4 | 94.3±10.7 | 91.8±9.4 | 0.999 | 0.805 | 0.765 |

PD-NC, Parkinson’s disease with no cognitive impairment; PD-MCI, Parkinson’s disease with mild cognitive impairment; PDD, Parkinson’s disease with dementia; UPDRS, Unified Parkinson’s Disease Rating Scale; GDS, Geriatric Depression Rating Scale.

The data are presented as mean ± SD.

^a^ Comparison between PD-NC and PD-MCI

^b^ Comparison between PD-MCI and PDD

^c^ Comparison between PD-NC and PDD

^d^ Off-state motor ratings according to the UPDRS (motor section).

^e^ Blood glucose was checked before injection of the radiopharmaceutical agent.
