# Supplementary material for: Cerebral Metabolic Differences Associated with Cognitive Impairment in Parkinson’s Disease
Source: PLoS One. 2016 Apr 11;11(4):e0152716. doi: 10.1371/journal.pone.0152716 (PMC4827825; doi:10.1371/journal.pone.0152716)
Supplement: S3 Table — (DOCX) [file pone.0152716.s003.docx]

**S3 Table. P values of Scheffe’s test for differences in neuropsychological scores between the PD groups.**

| **Cognitive test** | **PD-NC** | **PD-MCI** | **PDD** | **p Value^a^** | **p Value^b^** | **p Value^c^** |
| --- | --- | --- | --- | --- | --- | --- |
| **MMSE** | 28.5±1.7 | 28.4±1.3 | 23.2±2.3 | 0.979 | 0.000 | 0.000 |
| **Attenion and working memory** | | | |  |  |  |
| SDMT | 37.4±7.1 | 24.3±9.1 | 24.3±16.8 | 0.000 | 1.000 | 0.003 |
| TMT-A (s) | 57.8±14.3 | 71.2±19.9 | 96.7±53.8 | 0.219 | 0.052 | 0.001 |
| **Executive function** | | | |  |  |  |
| CWT-C time (s) | 69.7±13.3 | 84.7±24.3 | 122.4±37.9 | 0.031 | 0.000 | 0.000 |
| CWT-C right | 48.5±2.2 | 44.6±6.9 | 40.3±7.0 | 0.031 | 0.128 | 0.000 |
| TMT-B (s) | 152.0±34.6 | 200.8±61.3 | 245.8±91.7 | 0.016 | 0.129 | 0.000 |
| **Language** | | | |  |  |  |
| BNT | 24.6±3.2 | 22.3±2.7 | 21.1±3.9 | 0.046 | 0.672 | 0.017 |
| AFT | 17.1±3.5 | 16.2±3.3 | 13.3±3.1 | 0.620 | 0.099 | 0.012 |
| **Memory** | | | |  |  |  |
| AVLT-delay recall | 5.9±2.7 | 3.7±2.2 | 2.4±2.0 | 0.010 | 0.427 | 0.001 |
| AVLT-T | 29.7±10.1 | 22.7±7.4 | 13.7±6.8 | 0.028 | 0.039 | 0.000 |
| CFT-delay recall | 17.0±6.2 | 12.3±5.9 | 8.3±7.2 | 0.040 | 0.277 | 0.002 |
| **Visuospatial function** | | | |  |  |  |
| CFT | 34.3±2.1 | 30.6±5.6 | 21.7±13.4 | 0.144 | 0.003 | 0.000 |
| CDT | 23.0±5.1 | 18.4±6.7 | 11.4±8.6 | 0.046 | 0.023 | 0.000 |

PD-NC, Parkinson’s disease with no cognitive impairment; PD-MCI, Parkinson’s disease with mild cognitive impairment; PDD, Parkinson’s disease with dementia; MMSE, Mini Mental State Examination; SDMT, Symbol Digit Modality Test; TMT, Trail Making Test; CWT, Stroop Color-Word Test; BNT, Boston Naming Test; AFT, Animal Fluency Test; AVLT, Auditory Verbal Learning Test; CFT, the Rey-Osterrieth Complex Figure Test; CFT, Clock Drawing Test.

The data are presented as mean ± SD.

^a^ Comparison between PD-NC and PD-MCI

^b^ Comparison between PD-MCI and PDD

^c^ Comparison between PD-NC and PDD
